# Sleep‐disordered breathing, brain volume, and cognition in older individuals with heart failure

**DOI:** 10.1002/brb3.1029

**Published:** 2018-06-19

**Authors:** Chooza Moon, Kelsey E. Melah, Sterling C. Johnson, Lisa C. Bratzke

**Affiliations:** ^1^ College of Nursing University of Iowa Iowa City Iowa; ^2^ School of Nursing University of Wisconsin‐Madison Madison Wisconsin; ^3^ Wisconsin Alzheimer's Disease Research Center University of Wisconsin School of Medicine and Public Health Madison Wisconsin

**Keywords:** brain, gray matter, heart failure, sleep apnea syndrome, white matter

## Abstract

**Background and purpose:**

Sleep‐disordered breathing is common in individuals with heart failure and may contribute to changes in the brain and decreased cognition. However, limited research has explored how the apnea‐hypopnea index contributes to brain structure and cognition in this population. The aims of this study were to explore how the apnea‐hypopnea index is associated with brain volume and cognition in heart failure patients.

**Methods:**

Data of 28 heart failure patients (mean age = 67.93; SD = 5.78) were analyzed for this cross‐sectional observational study. We evaluated the apnea‐hypopnea index using a portable multichannel sleep‐monitoring device. All participants were scanned using 3.0 Tesla magnetic resonance imaging and neuropsychological tests. Brain volume was evaluated using a voxel‐based morphometry method with T1‐weighted images. We used multiple regressions to analyze how the apnea‐hypopnea index is associated with brain volume and cognition.

**Results:**

We found an inverse association between apnea‐hypopnea index scores and white matter volume (β = −0.002, *p *=* *0.026), but not in gray matter volume (β = −0.001, *p *=* *0.237). Higher apnea‐hypopnea index was associated with reduced regional gray and white matter volume (*p *<* *0.001, uncorrected). Cognitive scores were not associated with the apnea‐hypopnea index (*p*‐values were >0.05).

**Conclusion:**

Findings from this study provide exploratory evidence that higher apnea‐hypopnea index may be associated with greater brain volume reduction in heart failure patients. Future studies are needed to establish the relationship between sleep‐disordered breathing, brain volume, and cognition in heart failure samples.

## 1 INTRODUCTION

Heart failure (HF) patients present decreased brain volume (Bhattacharya et al., 2012; Woo, Macey, Fonarow, Hamilton & Harper, 2003). Approximately 50% of HF patients experience cognitive impairment in multiple domains including attention, memory, processing speed, and executive function (Pressler, 2008; Pressler et al., 2010; Vogels, Scheltens, Schroeder‐Tanka & Weinstein, 2007). One of the factors that may contribute to cognitive decline and brain changes in individuals with HF is sleep‐disordered breathing (SDB). Up to 75% of individuals with HF suffer from SDB, such as obstructive sleep apnea or central sleep apnea (Kasai, Floras & Bradley, 2012), which may lead to cognitive declines in attention, memory, visuospatial and constructional abilities, psychomotor speed, and executive function (Yaffe, Falvey & Hoang, 2014). Severe SDB, or higher apnea‐hypopnea index (AHI), is found to also be related to reductions in gray matter volume, increases in white matter hyperintensities, and increases in neural damage in general adult samples (Celle et al., 2016; Yaouhi et al., 2009; Zhang et al., 2013). However, existing research about brain structure in HF patients is limited, particularly testing the association between SDB, brain structure, and cognition (Hjelm, Strömberg, Arestedt & Broström, 2013; Knecht et al., 2012; Woo et al., 2003, 2015).

The purpose of this study was to explore how SDB, particularly obstructive sleep apnea, may contribute to cognition and brain volume in individuals with HF. The specific aims are (a) to explore how AHI is associated with decreases in global and regional, gray and white matter volume and (b) to explore the degree to which the AHI is associated with cognition. Our hypotheses were (a) a higher AHI is associated with decreased gray and white matter volume and (b) a higher AHI is associated with decreased cognition.

## 2 METHODS

This study was conducted at an academic university hospital in a Midwestern metropolitan city and used cross‐sectional, observational design. Data on 28 participants from the parent study were analyzed (Melah et al., 2016). To be eligible for the parent study, participants had to be 60 years or older, have a current diagnosis of HF confirmed by echocardiography, on optimized medical therapy, and have no major neurological or psychiatric disorders, stroke, current treatment for SDB, serious comorbid conditions (e.g., cancer and severe renal failure), or contraindications for MRI. We excluded 10 patients who are compliant to positive airway pressure treatment for SDB of 38 participants from the parent study because treatment may change the impact of AHI on brain volume (Kim, Joo, et al., 2016).

### 2.1 Procedures

The Institutional Review Board at the participating institution approved this study. Participants signed informed consent, and then, study staff sent questionnaires prior to the first study visit. At the study visit, research staff administered neuropsychological tests and each participant underwent an MRI. Participants were then asked to complete a one‐night, in‐home multichannel portable sleep‐monitoring test within a week of the study visit.

### 2.2 Apnea‐hypopnea index

To evaluate the AHI, we used an in‐home, multichannel portable sleep‐monitoring device called ApneaLink Plus (ResMed Corp, San Diego, CA, USA), which is a valid measure that has been used in HF samples (Hjelm et al., 2013; Sharma et al., 2017). The AHI is calculated and corresponds to the number of apneas and hypopneas in an hour. A certified clinician examined raw data to evaluate the quality and found that the ApneaLink Plus demonstrated a high sensitivity and specificity of 92.5% and 86%, respectively, at AHI ≥ 10 (de Vries et al., 2015). We chose an AHI of 10 as a cutoff to calculate the effect size, because an AHI of 10 is often used for clinical purposes and as a definition for suspected SDB using a multichannel portable sleep‐monitoring device (Collop et al., 2007; de Vries et al., 2015).

### 2.3 Sleep questionnaires

We used the Insomnia Severity Index (Morin, Belleville, Belanger & Ivers, 2011), Pittsburgh Sleep Quality Index (Buysse, Reynolds, Monk, Berman & Kupfer, 1989), and Epworth Sleepiness Scale (Johns, 1991) to evaluate insomnia symptoms, sleep quality, and excessive daytime sleepiness.

### 2.4 Neuropsychological tests

Comprehensive neuropsychological testing included measures within cognitive domains that have been shown to be impaired among people with HF, including attention, processing speed, memory, and executive functioning (Pressler et al., 2010). The battery included the Wechsler Memory Scale‐Revised Logical Memory subtest (Story A) (WMS‐R LM) (Wechsler, 1987), category fluency (animals, vegetables) (Rosen, 1980), the Wechsler Adult Intelligence Scale‐Revised Digit Span and Digit Symbol subtests (Wechsler, 1987), Trail Making Test (Reitan, 1985), and the Rey Auditory Verbal Learning Test (RAVLT) (Schmidt, 1996).

### 2.5 MRI acquisition

The MRI scans were conducted using the axial plane on a General Electric 3.0‐Tesla Discovery MR750 scanner (Waukesha, WI, USA) with a standard 8‐channel head coil. We used T1‐weighted volume with the following parameters: inversion time/echo time/repetition time = 450 ms/3.2 ms/8.1 ms, flip angle = 12°, slice thickness = 1.0 mm, field of view = 256 mm, and matrix size = 256 × 256. All scans were reviewed by a neuroradiologist for potential abnormalities. The study staff also visually reviewed all scans for quality assurance. None of the scans were excluded because of abnormalities or quality.

### 2.6 Global brain volume and intracranial volume

We used a “reverse brain masking” method (Keihaninejad et al., 2010) to calculate the global gray matter volume, global white matter volume, and intracranial volume (ICV) in Statistical Parametric Mapping (SPM) 8 (http://www.fil.ion.ucl.ac.uk/spm).

### 2.7 MRI processing for regional brain volume

Regions of gray matter volume and white matter volume were evaluated using voxel‐based morphometry (VBM) using the New Segment procedure in SPM8 (Good et al., 2001). First, each individual's T1 scan was segmented into gray matter, white matter, and cerebral‐spinal fluid tissue. Second, the probability maps were normalized to a Montreal Neurological Institute (MNI) template, using the Diffeomorphic Anatomical Registration Through Exponentiated Lie Algebra (DARTEL) normalization method (Ashburner, 2007) and resampled to 1.5‐mm cubic voxels. Then, a modulation step was completed to warp the image to the MNI template. These images were then smoothed using an 8‐mm isotropic Gaussian kernel (Ashburner, 2007; Good et al., 2001). To increase the anatomic plausibility, clusters were included using a threshold of >50 voxels.

### 2.8 Demographic variables and clinical variables

We reviewed the patients’ medical charts to gather demographic and clinical variables, such as age and sex, and clinical variables included the New York Association (NYHA) functional class, type of HF, left ventricular ejection fraction (LVEF, %), body mass index (BMI), and total comorbidity scores using the Charlson Comorbidity Index (Charlson, Pompei, Ales & MacKenzie, 1987).

### 2.9 Statistical analysis

Descriptive statistics were calculated to summarize the sample and multiple regression models to address Aims 1 and 2. To be consistent with findings from the previous literature regarding brain volume and cognition, we used the different set of covariates. The covariates were age, sex, ICV, and LVEF which is consistent with previous literature (Bhattacharya et al., 2012; Celle et al., 2016; Vogels, Oosterman, et al., 2007). To explore how the AHI is associated with cognition, we used separate hierarchical multiple regression models for each combination of raw scores of cognitive tests and the AHI. We included age, sex, education, comorbidity, and NYHA functional class as covariates based on previous reviews of the literature (Bauer, Johnson & Pozehl, 2011; Pressler et al., 2010). For all the analyses, SPSS version 22 (Chicago, IL, USA) was used, but SPM8 was used to assess the regional voxel‐wise level volume change. The Type I error rate was an alpha of 0.05 for all analyses. However, for voxel‐wise level analyses, we used an alpha of 0.001. Because our aims were only to explore, we did not correct for the Type I error rate, thereafter stated as “uncorrected.”

## 3 RESULTS

### 3.1 Comparison of the demographic and clinical factors

Table 1 summarizes demographic, clinical factors, and neuropsychological test scores. Global and regional brain volume reductions by changes in the AHI.

**Table 1:** Demographic and clinical information (*n *= 28)

	Mean ± SD or *n* (%)
Age	67.93 ± 5.78
Sex (Female)	17 (60)
Left ventricular ejection fraction	46.25 ± 12.39
Systolic heart failure	17 (61)
Diastolic heart failure	11 (40)
New York Heart Association functional class	
I	10 (36)
II	15 (53)
III	3 (11)
Charlson comorbidity index	1.11 ± 1.29
Body mass index	26.64 ± 4.15
Previous education	14.78 ± 2.41
Apnea‐hypopnea index	12.21 ± 10.28
Number of central sleep apnea events	3.07 ± 5.29
Pittsburgh sleep quality index	6.21 ± 3.72
Epworth sleepiness scale	6.93 ± 5.37
Insomnia severity index	5.07 ± 4.32
Sleep medications	4 (14)
RAVLT total trials 1–5	41.75 ± 8.39
RAVLT long delay free recall	28.21 ± 2.11
WMS‐R logical memory story A immediate recall	11.50 ± 3.44
WMS‐R logical memory story A delayed recall	9.36 ± 3.71
Trail making test A time (s)	30.00 ± 5.84
Trail making test B time (s)	94.12 ± 41.24
Animal fluency	20.00 ± 5.38
Vegetable fluency	12.82 ± 3.76
Digit span forward	8.29 ± 1.98
Digit span backward	6.96 ± 1.97
Digit span total score	15.25 ± 3.31
Digit symbol	45.63 ± 11.27
Intracranial volume (L)	1.51 ± 0.15
Gray matter volume (L)	0.61 ± 0.07
White matter volume (L)	0.41 ± 0.052

*Notes*

RAVLT, Rey auditory verbal learning test; WMS‐R, Wechsler memory scale‐revised logical memory.

### 3.2 Global volume

A higher AHI was not associated with a decrease in global gray matter volume, adjusting for covariates (β = −0.001, *p* = 0.237, Table S1). However, a higher AHI was associated with reduced global white matter volume (β = −0.002, *p* = 0.026, Table S1). Figure S1 is the scatter plot of the relationship between AHI and global gray and white matter.

### 3.3 Regional volume

**Figure 1:**
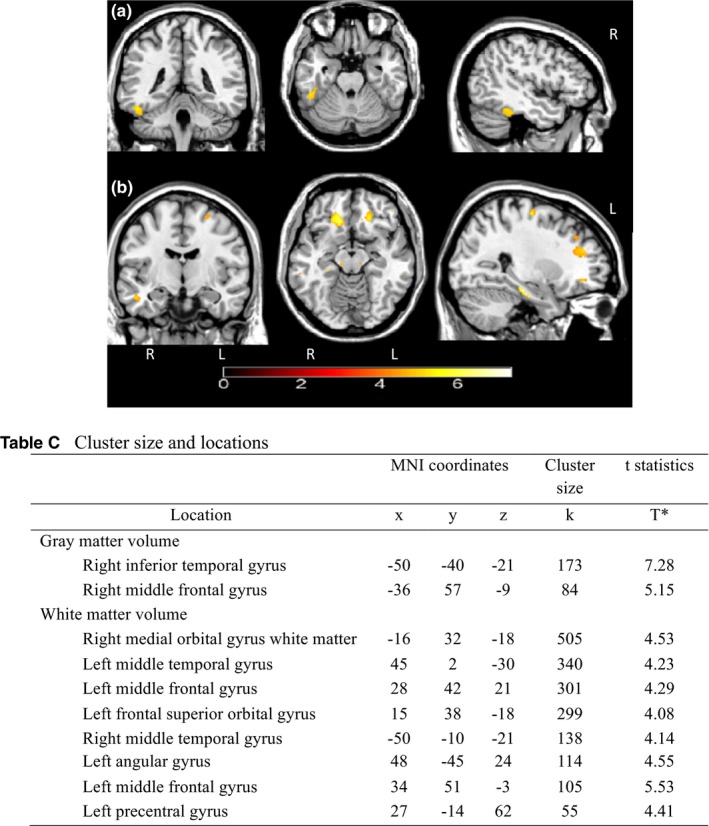
Areas of reduced brain volume by increases in the apnea‐hypopnea index (AHI). (a) Areas of reduced gray matter volume change by increases in apnea‐hypopnea index (b) Areas of reduced white matter volume change by increases in the apnea‐hypopnea index. The controlled for age, sex, intracranial volume, left ventricular ejection fraction in warm colors (yellow). The color bar indicates the *t* value. *p *< 0.001, uncorrected. Peak voxel coordinates, *t* values, *p* values, and cluster size are listed in Table C

Regional gray matter volume was reduced with an increase in the AHI, adjusting for the covariates (*p *<* *0.001, uncorrected). See Figure 1 for an example of the brain regions (A and B). The corresponding table shows the locations, statistics, and clusters. The regional white matter volume decreased with an increase in the AHI controlling for covariates (*p *<* *0.001, uncorrected). As in Figure 1a, gray matter volume reductions were located in the right inferior temporal gyrus and right middle frontal gyrus and white matter volume reductions were identified in the right medial orbital gyrus, right and left middle temporal gyrus, left middle frontal gyrus, and left frontal superior orbital gyrus (Figure 1b). Higher AHI was not associated with higher or lower gray matter or white matter volume. None of the voxels survived after correction for multiple comparisons.

### 3.4 The relationship between the AHI and neuropsychological tests

The AHI was not associated with any of the cognitive test scores among people who had not been diagnosed with SDB after controlling for covariates (*p *> 0.05, Table S2).

## 4 DISCUSSION

The findings from the current study provide exploratory evidence that severity of SDB may be associated with regional brain volume reduction among HF patients. To the best of our knowledge, this is the first study to document the relationship between SDB severity, brain volume, and cognition in a HF sample. We found an inverse association between AHI scores for global white matter volume, but not for global gray matter volume. AHI was associated with regional gray and white matter volume reduction. However, we did not find any association between AHI and cognition.

Regional reductions in regional gray and white matter volume were evident with increases in the AHI. In particular, we found more profound reductions in white matter volume compared to reductions in gray matter volume by AHI from global (white matter β = −0.002) and regional clusters. Given that our sample had fairly good sleep quality (mean = 6.2), it is more likely that changes in brain volume associated with hypoxia than sleep fragmentation. Although the magnitude of brain volume reduction by AHI changes in the models, given that β is only −0.002, intermittent hypoxia from SDB can lead to profound hemodynamic changes and oxygen saturation changes in the brain. These changes can prompt cerebral small vessel disease and blood–brain barrier dysfunction, which could increase white matter lesions, gray matter loss, and neuronal damage (Kim, Martinez, et al., 2015; Macey et al., 2008; Patel, Hanly, Smith, Chan & Coutts, 2015). SDB is found to be associated with attention, processing speed, and executive function, which are related to brain regions in prefrontal regions (Beebe & Gozal, 2002). Our findings are suggestive of white matter volume reduction in the frontal region, which aligns with previous conceptual framework of SDB and cognition.

We did not find any significant associations between AHI and cognitive scores, which contradict some previously reported findings in people without HF. Although the connection between SDB and cognition is more evident in adults without HF (Ju et al., 2012; Martin, Sforza, Roche, Barthélémy & Thomas‐Anterion, 2015), findings have been mixed in HF samples. Knecht et al. (2012) reported that SDB is related to worse performance on attention and executive function measures. However, Hjelm et al. (2013) did not find differences in cognition by SDB status in HF. Given that underlying hippocampal volume reduction and neurodegeneration could predict a future cognitive change, longitudinal studies may help researchers understand whether decreases in the brain volume could predict cognitive impairment in HF sample with higher risk of SDB (Bangen et al., 2018).

There are several limitations to note. First, we used a portable SDB‐monitoring device, which may not fully detect sleep architecture. However, ApneaLink plus is a validated tool that has been efficiently utilized in generalized settings with low cost in HF and non‐HF samples (Collop et al., 2007; Sharma et al., 2017). In‐lab polysomnography could provide additional information on SDB. Second, the study has a cross‐sectional design, so we cannot determine a causal relationship. Third, the study did not include a control group, which may provide a registration challenge. At last, we had a small sample size with preliminary findings. Given that prior imaging studies in HF are based on small sample sizes, our study adds to the literature by accounting for SDB in HF (Woo et al., 2003, 2015). Despite these caveats, the current study is among the first to examine how AHI is associated with brain volume and cognition to our knowledge.

This study preliminarily supports that severity of SDB is associated with reduced brain volume in brain regions but not cognition among patients with HF. Future studies could address this topic using a larger sample size with a longitudinal design. The findings from our study provide information for researchers to better understand the potential etiological underpinnings of cognitive impairment and potentially modifiable causes of cognitive impairment in HF patients. Because SDB could also impact cerebral blood flow and axonal tracts in the brain, researchers could investigate 4D blood flow and diffusion tensor imaging to better understand brain structures in depth (Rivera‐Rivera et al., 2017). Clinicians could use this information to emphasize the importance of managing SDB to prevent further brain alterations.

## DISCLOSURE STATEMENT

The project described in this study was supported by the Sigma Theta Tau Beta‐Eta at Large chapter and the National Institute of Nursing Research, award Number R00NR012773 (Brain Alterations and Cognitive Impairment in Older Adults with Heart Failure). The content is solely the responsibility of the authors and does not necessarily represent the official views of the National Institute of Nursing Research or the National Institutes of Health. The authors have indicated no financial conflict of interest.

## AUTHOR CONTRIBUTIONS

Chooza Moon, PhD (Corresponding author), contributed to study design, data acquisition, analysis, interpretation, and writing the manuscript. Kelsey E. Melah, BS, contributed to data acquisition and data analysis. Sterling C. Johnson, PhD, contributed to magnetic resonance imaging data acquisition, data analysis, and revision of the manuscript. Lisa C. Bratzke, PhD, RN, ANP‐BC, contributed to study design, data acquisition, and revision of the manuscript.
