# Supplementary material for: Sleep‐disordered breathing, brain volume, and cognition in older individuals with heart failure
Source: Brain Behav. 2018 Jun 19;8(7):e01029. doi: 10.1002/brb3.1029 (PMC6043704; doi:10.1002/brb3.1029)
Supplement: Supplementary file 1 [file BRB3-8-e01029-s001.pdf]

Appendix. Figure A. Gray and white matter volume and apnea hypopnea index

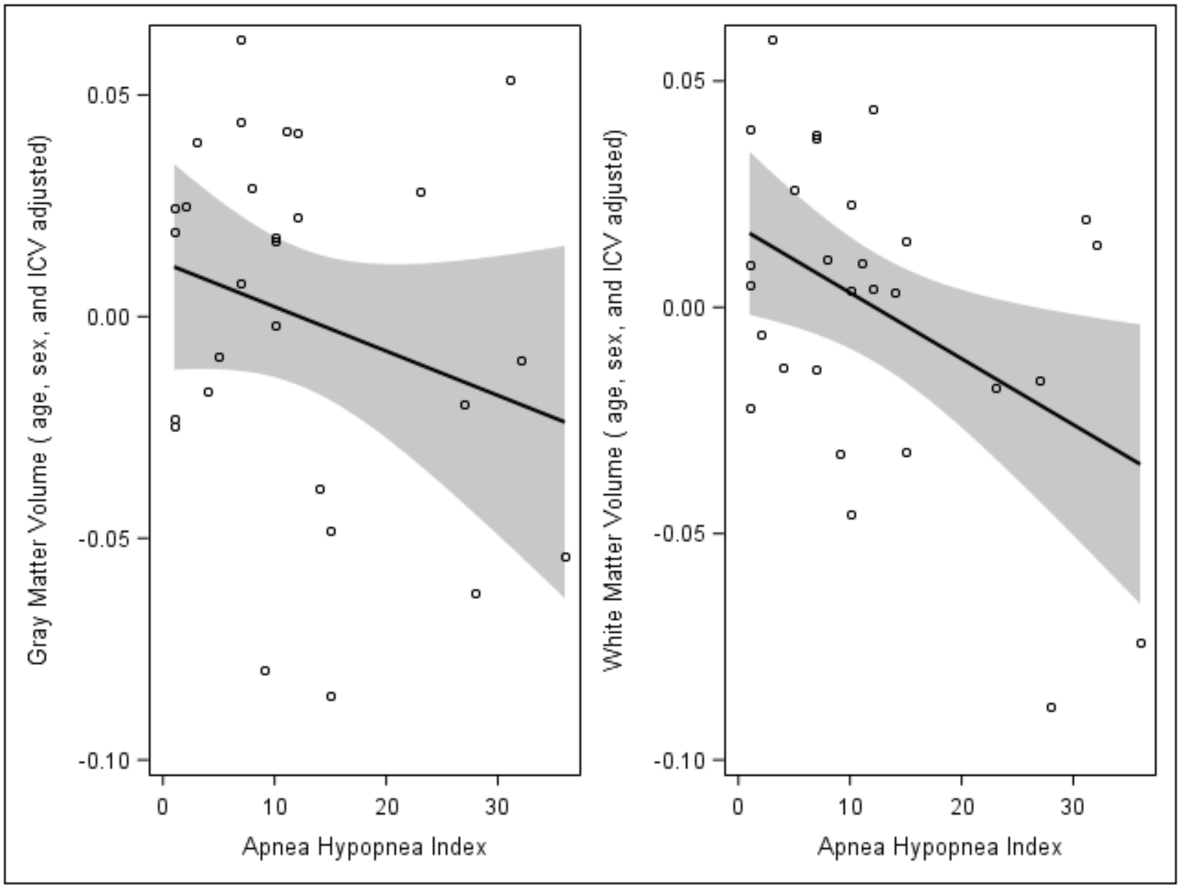

ICV = Intracranial volume.
