# Supplementary material for: Sleep‐disordered breathing, brain volume, and cognition in older individuals with heart failure
Source: Brain Behav. 2018 Jun 19;8(7):e01029. doi: 10.1002/brb3.1029 (PMC6043704; doi:10.1002/brb3.1029)
Supplement: Supplementary file 2 [file BRB3-8-e01029-s002.docx]

| Appendix. Table A. Summary of regression analysis for variables predicting brain volume with changes in apnea hypopnea index (AHI) (n=28) | | | | | |
| --- | --- | --- | --- | --- | --- |
|  | B | SE (B) | *β* | ΔR^2^ | R^2^/Adjusted R^2^ |
| Dependent variable : Gray matter volume | | | | | |
|  |  |  |  |  | 0.693/0.601 |
| Intercept | 0.653 | 0.157 |  |  |  |
| Age | -0.007 | 0.001 | -0.558 | 0.389* |  |
| Sex | 0.009 | 0.021 | 0.062 | 0.039 |  |
| ICV | 0.000 | 0.000 | 0.545* | 0.227* |  |
| LVEF | 0.001 | 0.001 | 0.118 | 0.008 |  |
| AHI | -0.001 | 0.001 | -0.183 | 0.029 |  |
| Dependent variable : White matter volume | | | | | |
|  |  |  |  |  | 0.669/0.570 |
| Intercept | -0.137 | 0.122 |  |  |  |
| Age | -0.002 | 0.001 | -0.199 | 0.080 |  |
| Sex | 0.016 | 0.017 | 0.151 | 0.059 |  |
| ICV | 0.000 | 0.000 | 0.762* | 0.442* |  |
| LVEF | 0.000 | 0.001 | -0.053 | 0.009 |  |
| AHI | -0.002 | 0.001 | -0.310* | 0.085* |  |
| AHI = Apnea hypopnea index; ICV= Intracranial volume  Note: age was centered to the mean value.  **p < .05* | | | | | |
