# Supplementary material for: Sleep‐disordered breathing, brain volume, and cognition in older individuals with heart failure
Source: Brain Behav. 2018 Jun 19;8(7):e01029. doi: 10.1002/brb3.1029 (PMC6043704; doi:10.1002/brb3.1029)
Supplement: Supplementary file 3 [file BRB3-8-e01029-s003.docx]

Appendix . Table B. Summary of coefficients when testing how apnea hypopnea index (AHI) was associated with cognitive test scores with regression models (n = 28)

| Dependent variables on each regression analysis | B | SE (B) | *β* | ΔR^2^ |
| --- | --- | --- | --- | --- |
| RAVLT total trials 1-5 | 0.077 | 0.130 | 0.110 | 0.011 |
| RAVLT long delay free recall | -0.021 | 0.045 | -0.101 | 0.010 |
| WMS-R logical memory story A immediate recall | 0.010 | 0.061 | 0.029 | 0.001 |
| WMS-R logical memory story A delayed recall | -0.063 | 0.064 | -0.179 | 0.030 |
| Trail making test A time | -0.361 | 0.201 | -0.335 | 0.105 |
| Trail making test B time | 0.163 | 0.863 | 0.041 | 0.001 |
| Animal fluency | 0.031 | 0.114 | 0.061 | 0.003 |
| Vegetable fluency | 0.075 | 0.051 | 0.217 | 0.044 |
| Digit span forward | 0.011 | 0.036 | 0.058 | 0.003 |
| Digit span backward | 0.015 | 0.040 | 0.085 | 0.007 |
| Digit span total score | 0.026 | 0.059 | 0.086 | 0.007 |
| Digit symbol | 0.006 | 0.161 | 0.006 | 0.000 |

RAVLT = Rey Auditory Verbal Learning Test; WMS-R = Wechsler Memory Scale-Revised Logical Memory

Note: AHI was not a significant factor related to any of the dependent variables; Covariates in the models included age, sex, education, comorbidities, and New York Heart Association functional class.
